# P-658. Emergence and Clade Expansion of Human Adenovirus B3 in Pediatric SARI: A Comparative Multicenter Study Between Brazil and Colombia, 2022–2023

**DOI:** 10.1093/ofid/ofaf695.871

**Published:** 2026-01-11

**Authors:** Sonia Mara Raboni, Maria Angelica Maya Restrepo, Heloisa Giamberardino, Jaime Usuga, Meri Nogueira, Ana Luisa Giamberardino, Luciane Pereira, Laura Ferreira, Vivian Torrecilha, Francisco Averhoff, Paulina Rebolledo, Gavin Cloherty, Michael G Berg, J O R G E OSORIO, Juan P Hernandez-Ortiz

**Affiliations:** Federal University of Paraná, Curitiba, Parana, Brazil; Universidad Nacional de Colombia, Medellín, Antioquia, Colombia; Hospital Pequeno Príncipe, Curitiba, Parana, Brazil; GHI One Health Colombia, Universidad Nacional de Colombia, Medellin, Colombia, Medellin, Antioquia, Colombia; Universidade Federal do Paraná, Curitiba, Parana, Brazil; Hospital Pequeno Príncipe, Curitiba, Parana, Brazil; Universidade Federal do Paraná, Curitiba, Parana, Brazil; Universidade Federal do Paraná, Curitiba, Parana, Brazil; Universidade Federal do Paraná, Curitiba, Parana, Brazil; Abbott Laboratories, Atlanta, Georgia; Emory University School of Medicine, Emory University Rollins School of Public Health, Atlanta, GA; Abbott, Abbott Park, Illinois; Abbott Labs, Abbott Park, Illinois; Global Health Institute, Madison, Wisconsin; Universidad Nacional de Colombia, Medellín, Antioquia, Colombia

## Abstract

**Background:**

Between 2022–2023, surveillance networks in Curitiba (Brazil) and Antioquia (Colombia) reported increased incidence and altered seasonality of respiratory tract infections. This study compared clinical and epidemiologic features, outcomes, and genotypes of patients with HAdV-associated SARI before and during outbreaks in both regions.

**Figure 1.**
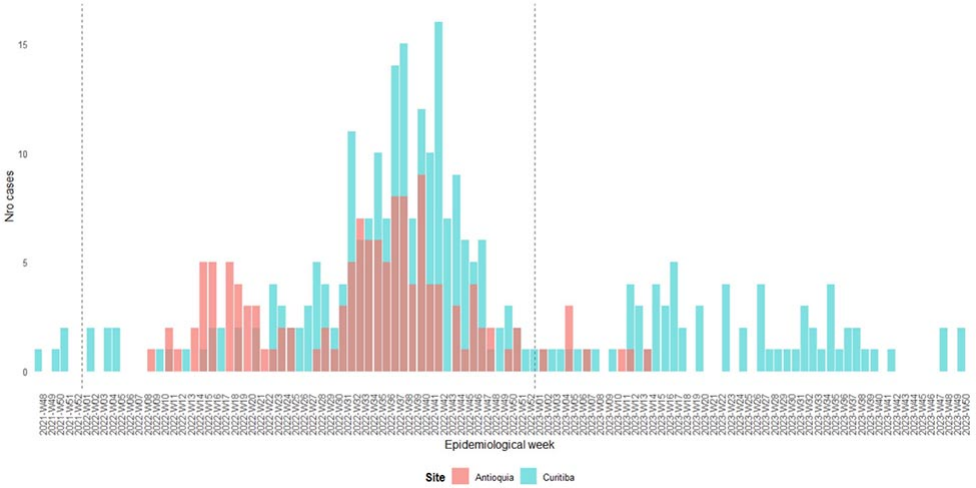
Distribution of HAdV-positive patients by epidemiological week according to consultation day in Curitiba (Brazil) and Antioquia (Colombia). Curitiba (green bars) and Antioquia (red bars).

**Figure 2.**
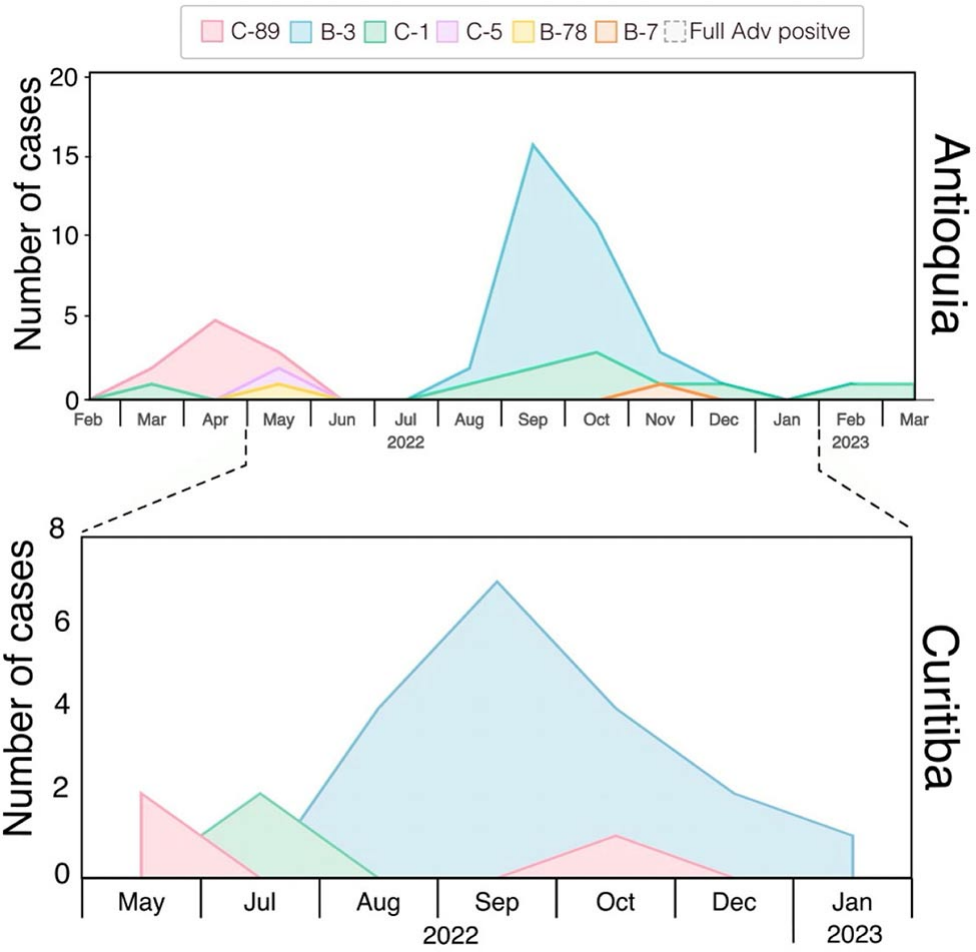
Distribution of HAdV Genotypes in Antioquia (Colombia) and Cutitiba (Brazil), 2022 - 2023 A. Distribution of various HAdV genotypes in Antioquia from February 2022 to March 2023, B. Distribution of various HAdV genotypes in Curitiba (Brazil) from May 2022 to January 2023. Areas represent the percentage prevalence of strains obtained in each period: HAdV-C89 (red), HAdV-C1 (green), HAdV-C5 (purple), HAdV-B3 (blue), HAdV-B78 (yellow), HAdV-B7 (orange). A. number of sequences: 54 samples. B. Number of sequences: 12 samples

**Methods:**

We conducted a multicenter observational study in pediatric patients with SARI and confirmed HAdV by PCR. In Antioquia, samples were collected from four hospitals (Feb 2022–Apr 2023); in Curitiba, samples were obtained from patients admitted to the Pequeno Príncipe Hospital (PPH) and the University Hospital of the Federal University of Paraná (CHC/UFPR) (Dec 2021–Dec 2023). Selected samples underwent Illumina sequencing. Clinical and epidemiologic data were extracted from records. Comparative analyses and phylogenetic/SNP analyses were performed to assess temporal dynamics and genetic diversity.

**Figure 3.**
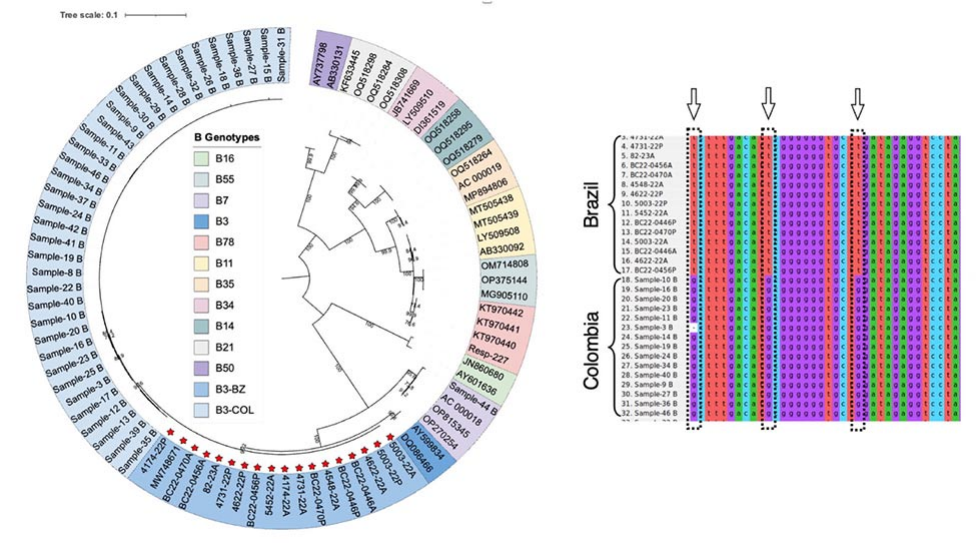
Phylogenetic and SNP Analysis of HAdV-B3 Strains Circulating in Curitiba and Antioquia, 2022–2023 The three nucleotides that differentiated the two clades are surrounded by discontinuous lines. B3-COL represents the samples collected in Antioquia (light blue), and B3-BZ represents the samples collected in Curitiba (dark blue).

**Table 1.**
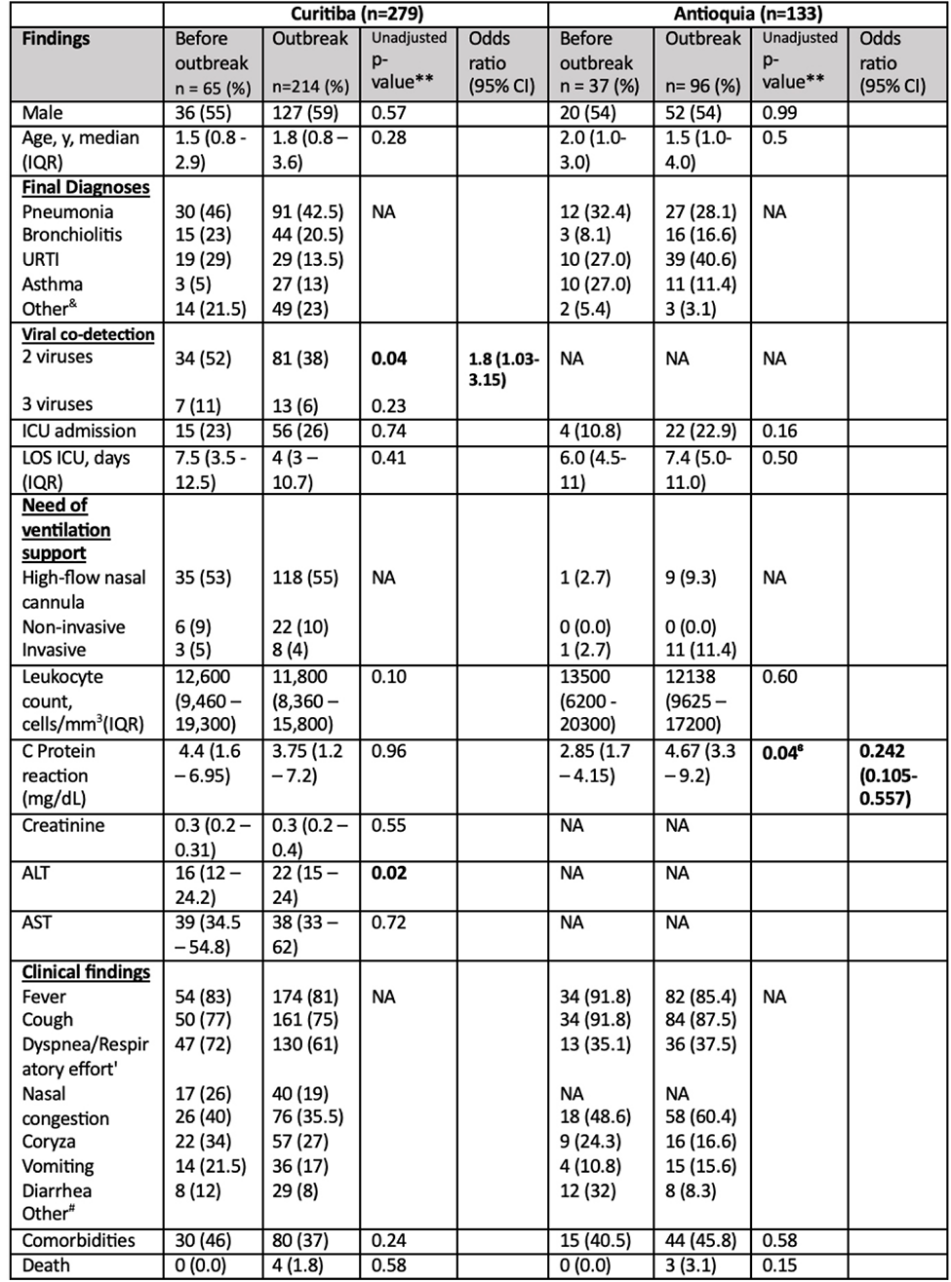
Sociodemographic, Clinical, and Laboratory Characteristics of Adenovirus-Positive Patients with SARI in Curitiba and Antioquia: Pre- and Post-Outbreak Comparison

**Results:**

In 2022, HAdV-associated SARI cases rose after EW20 in Curitiba and EW26 in Antioquia, peaking at EW41 and EW39, respectively. The outbreak ended in Antioquia by EW51/2022; Curitiba reported elevated cases through EW35/2023 (Figure 1). HAdV-B3 emerged post periods dominated by HAdV-C1/C89. SNP analysis showed two distinct B3 clades: B3-COL and B3-BZ, separated by 3 nucleotide differences (Figure 3). Curitiba identified 279 cases (214 outbreak-related); Antioquia reported 133 cases (96 outbreak-related). No significant demographic or clinical differences were found across periods or regions. In Curitiba, the most commonly detected viruses were rhinovirus, RSV, and metapneumovirus.

**Conclusion:**

The HAdV-associated SARI outbreak began earlier in Curitiba than in Antioquia. In both locations, HAdV-C predominated during the first half of 2022, and HAdV-B3 became the predominant genotype during the outbreak in the second half of 2022 and the first months of 2023. Phylogenetic analysis revealed that the HAdV-B3 strains circulating in Curitiba and Antioquia shared a most recent common ancestor (tMRCA), leading to the formation of two distinct clades. Phylogenetic analysis suggests the B3-COL clade likely originated from B3-BZ clade.

**Disclosures:**

All Authors: No reported disclosures

